# The Effects of Extracorporeal Shock Wave Therapy in Patients with Coccydynia: A Randomized Controlled Trial

**DOI:** 10.1371/journal.pone.0142475

**Published:** 2015-11-10

**Authors:** Shih-Feng Lin, Yi-Jen Chen, Hung-Pin Tu, Chia-Ling Lee, Ching-Lin Hsieh, Wen-Lan Wu, Chia-Hsin Chen

**Affiliations:** 1 Department of Physical Medicine and Rehabilitation, Kaohsiung Municipal Ta-Tung Hospital, Kaohsiung, Taiwan; 2 Department of Physical Medicine and Rehabilitation, Kaohsiung Medical University Hospital, Kaohsiung, Taiwan; 3 Graduate Institute of Clinical Medicine, College of Medicine, Kaohsiung Medical University, Kaohsiung, Taiwan; 4 Department of Public Health and Environmental Medicine, School of Medicine, College of Medicine, Kaohsiung Medical University, Kaohsiung, Taiwan; 5 Department of Physical Medicine and Rehabilitation, Kaohsiung Municipal Hsiao-Kang Hospital, Kaohsiung, Taiwan; 6 School of Occupational Therapy, College of Medicine, National Taiwan University, Taipei, Taiwan; 7 Department of Sports Medicine, College of Medicine, Kaohsiung Medical University, Kaohsiung, Taiwan; 8 Department of Physical Medicine and Rehabilitation, School of Medicine, College of Medicine, Kaohsiung Medical University, Kaohsiung, Taiwan; Stavanger University Hospital, NORWAY

## Abstract

Coccydynia is pain in the coccygeal region, and usually treated conservatively. Extracorporeal shock wave therapy (ESWT) was incorporated as non-invasive treatment of many musculoskeletal conditions. However, the effects of ESWT on coccydynia are less discussed. The purpose of this study is to evaluate the effects of ESWT on the outcomes of coccydynia. Patients were allocated to ESWT (n = 20) or physical modality (SIT) group (n = 21) randomly, and received total treatment duration of 4 weeks. The visual analog scale (VAS), Oswestry disability index (ODI), and self-reported satisfaction score were used to assess treatment effects. The VAS and ODI scores were significantly decreased after treatment in both groups, and the decrease in the VAS score was significantly greater in the ESWT group. The mean proportional changes in the ODI scores were greater in the ESWT group than in the SIT group, but the between-group difference was not statistically significant. The patients in the ESWT group had significantly higher subjective satisfaction scores than SIT group. We concluded that ESWT is more effective and satisfactory in reducing discomfort and disability caused by coccydynia than the use of physical modalities. Thus, ESWT is recommended as an alternative treatment option for patients with coccydynia.

**Trial Registration:**

ClinicalTrials.gov NCT02313324

## Introduction

Coccydynia is pain around the coccygeal region that may be caused by sudden impact over the coccyx area from falls or traumatic injuries, resulting in pain and inflammatory changes of the surrounding ligaments and muscles [1]. Pain in these conditions is associated with coccygeal instability or subluxation, and patients develop subsequent coccydynia [2]. The radiographic orientations of the coccyx were introduced by Postacchini and Massobrio [3], who observed that patients with symptoms of coccydynia showed more mobility over the first intercoccygeal joint. The pain drawings were used to identify the correct symptoms and offer valuable methods for relieving the symptoms of coccydynia.

Patients with coccydynia often complain of pain and local tenderness around the coccyx [4]. Surgical interventions with the excision of the mobile coccyx or a total coccygectomy relieve approximately 80% to 90% of symptoms [5]. The most common complication of coccygectomy is wound infection; therefore, the surgery is rarely performed, and nonsurgical strategies remain the major treatment for coccydynia. Management typically includes medications such as nonsteroidal anti-inflammatory agents (NSAIDs), gentle massage over the ligaments attached to the sacrococcygeal joint [6], manual manipulation for mal-alignment of the coccyx [7,8], local steroid injections combined with anesthesia [9], and physical therapy with interferential current (IFC) [10] or shortwave diathermy (SWD) [11]. SWD is used to provide heat to deep tissues [12] and is useful in relieving pain and muscle spasms in inflammatory tissue [13]. SWD is regarded as an effective treatment modality for chronic osteoarthritis [14] and is used for somatic pain generated by the ligamentous and muscular elements inserted into the coccyx [15]. SWD can be effective for the treatment of patients with chronic low back pain [16] and was reported to reduce pain in patients with coccydynia [17]. IFC has been reported to reduce inflammation-induced central sensitization [18], and reduce low back pain [19,20]. Combined with other physical modalities, IFC showed better outcomes in reducing the pain intensity associated with musculoskeletal disorders [10].

In recent years, extracorporeal shock wave therapy (ESWT) has been suggested for non-invasive treatment of many musculoskeletal conditions, including plantar fasciitis [21], epicondylitis [22] and shoulder calcification [23]. The effectiveness of pain relief with ESWT might be due to stimulation analgesia [24] and increased tissue regeneration [25]. However, the effects of ESWT on low back pain and coccydynia are less discussed till now. Lee et al. reported exercise program combined with ESWT relieved chronic low back pain and improved dynamic balance more than exercise program with conservative physical therapy [26]. There is only one recent report by Marwan et al., presenting 2 cases with coccydynia, and reported three sessions of ESWT effective in relieving pain, and the pain did not recur during one year follow-up period [27]. Most patients prefer conservative treatment with non-invasive methods for coccydynia, including physical modalities and ESWT. There are few studies comparing the effects between ESWT and SWD combined with IFC therapy (SIT) in patients with coccydynia. The purpose of this study is to evaluate the effects of non-invasive ESWT on the outcomes of coccydynia.

## Materials and Methods

### Study population

**Fig 1:**
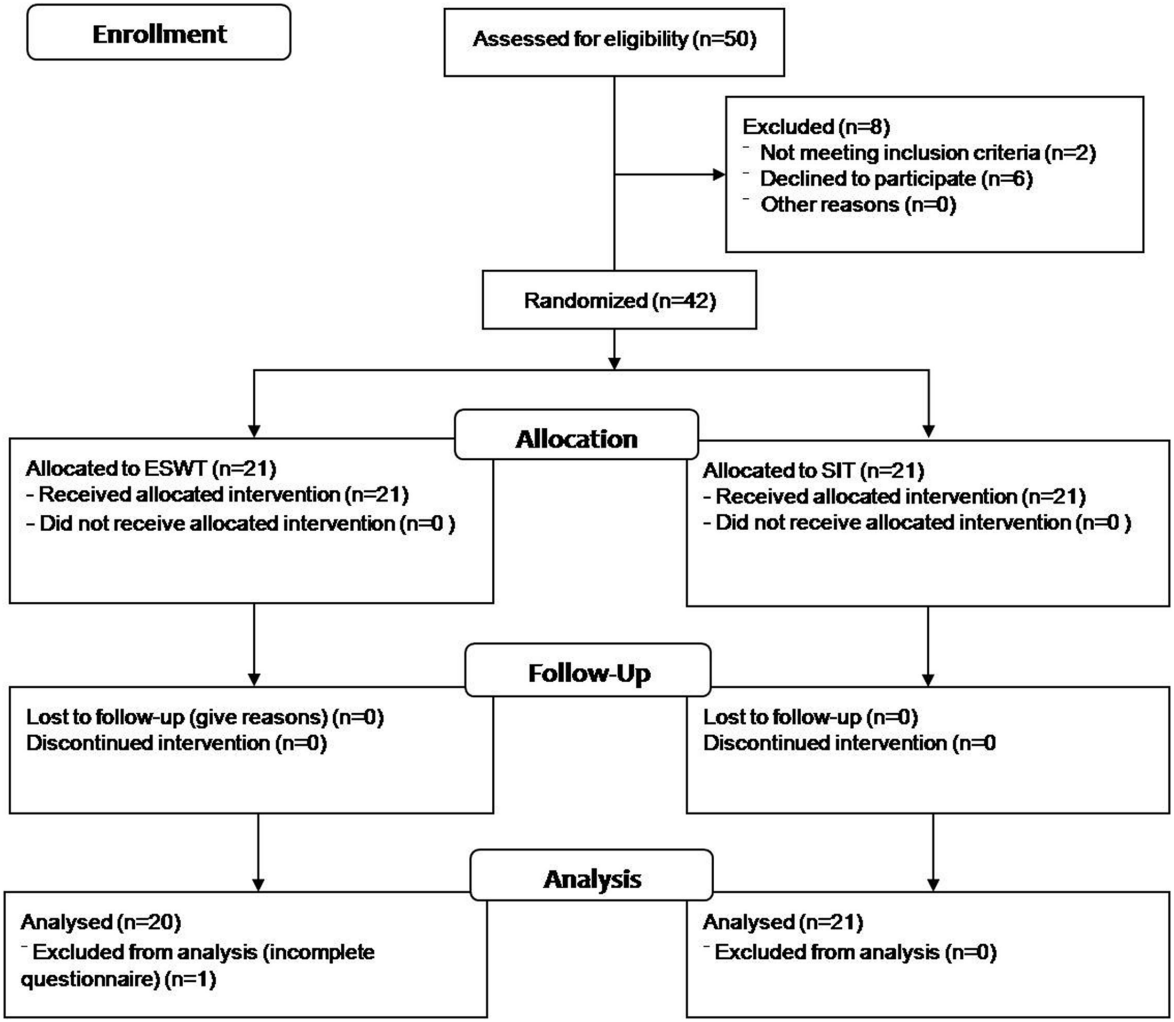
Flow diagram of the enrollment.

This clinical study was approved by the Institutional Review Board (IRB) of Kaohsiung Medical University Hospital (IRB number: KMUH-IRB-20120218). The study was carried out from November, 2012 to November 2013 according to the IRB approval of the study period. This clinical study was conducted after the approval of IRB for its study design and ethical concerns, without initial registration as clinical trial, since researchers can start recruiting subjects and executing clinical studies once the clinical studies are approved by the IRB. The study was later registered on ClinicalTrials.gov Protocol and Results Registration System (registration number: NCT02313324) as suggested. The authors confirmed that all ongoing and related trials for this intervention are registered. The CONSORT checklist for randomized trial is included in Supporting Information (S1 Text). The patients with first-time diagnosis of coccydynia with pain score ranging from 1 to 10 were enrolled at the rehabilitation outpatient department (OPD). Patients with a history of direct traumatic events to the buttocks, such as falls or slipping, were also included. The physiatrists of rehabilitation OPD were responsible for patient enrollment. A total of 50 participants were screened. After providing informed consent in written form, the patients’ histories were taken, and they underwent physical examination to identify the tender area and bony position. Roentgenography was used to assess the coccyx position and rule out major coccyx dislocations. Patients with cardiac pacemaker, tumors of the cauda equina, pelvic surgery, herniation of the lumbosacral disc, internal procidentia, genitourinary or gastrointestinal complaints or psychogenic factors were excluded. The basic demographic features and duration of coccygeal pain were recorded. Finally, 42 patients met the inclusion criteria, and were randomly allocated to 2 groups with allocation ratio of 1:1. The CONSORT diagram was shown in Fig 1. The physiatrist in charge of randomization was not involved in the study assessment or data analysis.

### Evaluation

Before treatment, all of the patients reported the pain intensity, using a visual analog scale (VAS), on a scale from 0 through 100 mm (0 for no pain and 100 mm for the worst pain) [28]. Disability and pain were measured using a spinal disorder-specific questionnaire, the Oswestry disability index (ODI, in Chinese) [29]. There are six statements to be ranked on a scale of 0 to 5 in each of the 10 sections of the questionnaire. The contents relate to impairments such as pain intensity and abilities in personal care, lifting, walking, sitting, standing, sleeping, sex life, social life and traveling. The patients were asked to identify the corresponding level of disability in each section. The total score ranges from 0 to 50; 0 represents the highest level of function, and 50 indicates completely disabled. In addition to the evaluations of the VAS and ODI prior to treatment, the scores were followed at the 5^th^ and 8^th^ week post-enrollment follow-up visits. Self-reported satisfaction scales were used to assess the general condition at the 8^th^ week.

### Interventions

The patients were randomly allocated to two parallel groups sequentially in a 1:1 ratio, the SIT group and the ESWT group, at the initial OPD visit. The enrolled patients were asked to discontinue analgesics or other physical modalities for pain one week prior to and after the treatments. The patients in the SIT group received combined therapy with SWD and IFC performed by the same physiotherapist at each treatment session. The SWD treatments followed the method described in our previous study [8]. The SWD (Cosmogamma, Italy, SW500) mode was the inductive mode with a coil at a frequency of approximately 27.12 MHz. The patients were maintained in the prone position with two pillows under the abdominal area, and the sacrococcygeal area was covered with a towel to absorb sweat. The SWD applicator was placed over the sacroccygeal area. The treatment duration was 20 minutes. After completing the SWD treatments, the patients received the IFC (Minato, Japan) treatment. IFC provides deeper electrical stimulation. For this treatment, the skin overlying the sacral area was wiped with alcohol, and the electrical current was applied to the gluteal area using four electrodes from 2 channels of the stimulator. The four electrodes were set in a therapeutic way such that the two channels crossed each other with specific regions of interest concentrated on the gluteal area. The carrier frequency, typically 4000 Hz and 4100 Hz and designed to interfere with each other, resulted in a beat frequency of 100 Hz within the treated area. The treatment duration was 20 minutes. The protocol was set as 3 times a week for a period of 4 weeks.

The ESWT was performed by the same physiatrist, who was blinded to patients’ enrollment, and was not involved in further follow-up assessment. A BTL-5000 radial ESWT with a median head applicator was used (BTL Industries Inc. Columbia, South Carolina, USA). The patients were maintained in the prone and knees separated position with a pillow under the abdominal area. The patients received 2000 shots of ESWT in the coccyx area per session for four sessions (one session a week for 4 consecutive weeks). The frequency used was 5 Hz and the pressure was 3–4 bar. The detailed descriptions of the study protocol are included in the Supporting Information, both in original and translated forms (S2 and S3 Texts).

### Statistical analysis

The results were assessed by another therapist who was blinded to the patient’s treatment method. All of the statistical analyses were performed by a statistician. We calculated the sample size of 15 patients per group with the assumption of a mean pain intensity difference between the two groups of 20 mm (SD 16), which provided 90% power to detect such a difference with a two-sample *t* test and a two-sided type I error of 0.05. Therefore we aimed to recruit 40 patients (20 per group) in our study. Power analysis and sample size determination were performed using the G*Power version 3.1.92 [30]. Characteristics of the study participants for the continuous and categorical variables were analyzed by *t* test, the Wilcoxon rank-sum test, and the chi-squared test, as appropriate, for comparisons between the ESWT and SIT groups. The clinical evaluation was designed as a repeated measure, and werepeated measure, and we repeated measure, and we repeated measure, and we used random (PROC GLIMMIX) statements to account for a period of 8 weeks taken from the same individual between the ESWT and SIT groups. Therefore, the repeated measures data were analyzed with the quantitative data (VAS and Therefore, the repeated measures data were analyzed with the quantitative data (VAS and ODI) by a by a generalized linear mixed model ((PROC GLIMMIX statement), and conclusions were drawn according to the parameter (β, regression coefficient) and difference means statement), and conclusions were drawn according to the parameter (â, regression coefficient) and difference means with Dunnett’s multiple-comparison post hoc test inin the ESWT and SIT groups. We next examined the interactions between ESWT and duration for VAS and. We next examined the interactions between ESWT and duration for VAS and ODI using repeated-measure using repeated-measure generalized linear mixed models. Differences between the ESWT and SIT groups in the subjective satisfaction score at the 8^th^ week follow-up were analyzed by *t*-test / Wilcoxon rank-sum test. The significance level was set at 0.05 by two-tailed tests. The analyses were performed using SAS version 9.3 statistical software (SAS Institute Inc, Cary, North Carolina, USA).

## Results

### Patient characteristics

A total of 42 patients were recruited. The enrolled patients were equally allocated to ESWT and SIT groups, with 21 patients in each allocation. One patient in the ESWT group was excluded from analysis due to incomplete information from the questionnaire. There were eventually 20 patients in the ESWT group, and 21 patients in the SIT group. The basic characteristics of the included patients were shown in Table 1. There were no between-group differences in the age, gender, and onset duration prior to treatment.

**Table 1:** Baseline characteristics of the included patients.

	ESWT (N = 20)	SIT (N = 21)
Age, yrs	44.75(14.85)	44.46(18.88)
Gender, female, n (%)	15(75.0)	15 (71.43)
BMI, kg/m^2^	24.22(5.57)	22.45(3.12)
Time since onset, months	10.51(13.05)	12.57(16.48)

BMI: body mass index; ESWT: extracorporeal shock wave therapy; SIT: physical modality.

### Clinical outcomes

The VAS score decreased by 30.5 mm and 41.0 mm in the ESWT group at the 5^th^ and 8^th^ week post-treatment evaluations, respectively (p<0.001). In the SIT group, the VAS scores decreased by 16.2 mm and 21.0 mm at the 5^th^ and 8^th^ week post-treatment evaluations, respectively (p<0.001). The decrease in the VAS score was significantly greater in the ESWT group compared with the SIT group at the 5^th^ week evaluation (p = 0.004, Table 2); the between-group difference was more significant at the 8^th^ week assessment. Differences between 5^th^ week assessment and 8^th^ week assessment in ESWT group were -10.5 mm (p<0.05) and differences in SIT group were -4.8 mm (p>0.05) with Dunnett’s multiple-comparison post hoc test. Calculating the proportional change in the VAS score following the formula below, we found that the mean changes at the 5^th^ week assessment and 8^th^ week assessment were greater in the ESWT group (-49.19% to -66.13%) than in the SIT group (-26.77% to -34.66%). The mean proportional change in the VAS score in the ESWT group was 31.47% greater than in the SIT group (66.13%−34.66% = 31.47%).

**Table 2:** The visual analog scale (VAS) at baseline, 5^th^ week, and 8^th^ week.

	ESWT (N = 20)	SIT (N = 21)	P value
VAS (mm)			
Initial assessment (SD)	62.00(18.24)	60.48(16.87)	0.783
5^th^ week assessment (SD)	31.50(18.99)	44.29(19.89)	0.042
8^th^ week assessment (SD)	21.00(18.32)	39.52(24.39)	0.009
Repeated measures^a^, β, P-value	-20.50, < 0.001	-10.48, 0.005	
ESWT vs SIT, P for interaction	0.004		

SD: standard deviation; ESWT: extracorporeal shock wave therapy; SIT: physical modality.

^a^ β, P-value and interaction by generalized linear mixed model; PROC GLIMMIX, repeated-measure.repeated-measure.

Proportional change in VAS score (%) = 100 × (5^th^ or 8^th^ week assessment—Initial assessment) / Initial assessment

There were significant decreases in the ODI scores at the 8^th^ week post-treatment evaluations in the ESWT and SIT groups (p<0.001 and p = 0.015, respectively, Table 3). The changes in the ODI score were not significantly different between the groups (p>0.05). Calculating the proportional change in the ODI score following the formula below, we found that the mean changes at the 5^th^ week assessment and 8^th^ week assessment were greater in the ESWT group (-41.31% to -60.51%) than in the SIT group (-43.08% to -49.00%). The mean proportional change in the ODI score in the ESWT group was 11.51% greater than in the SIT group (60.51%−49.00% = 11.51%).

**Table 3:** The Oswestry disability index (ODI) at baseline, 5^th^ week, and 8^th^ week.

	ESWT (N = 20)	SIT (N = 21)	P value
ODI			
Initial assessment (SD)	25.78 (14.14)	36.35 (17.42)	0.040
5^th^ week assessment (SD)	15.13 (9.09)	20.69 (21.33)	0.289
8^th^ week assessment (SD)	10.18 (10.85)	18.54(22.35)	0.139
Repeated-Measures β, P-value	-7.80, <0.001	-8.90, 0.015	0.331
ESWT vs SIT, P for interaction	0.742		

SD: standard deviation; ESWT: extracorporeal shock wave therapy; SIT: physical modality.

Proportional change in ODI score (%) = 100 × (5^th^ or 8^th^ week assessment—Initial assessment) / Initial assessment

### Subjective satisfaction of the patients

**Fig 2:**
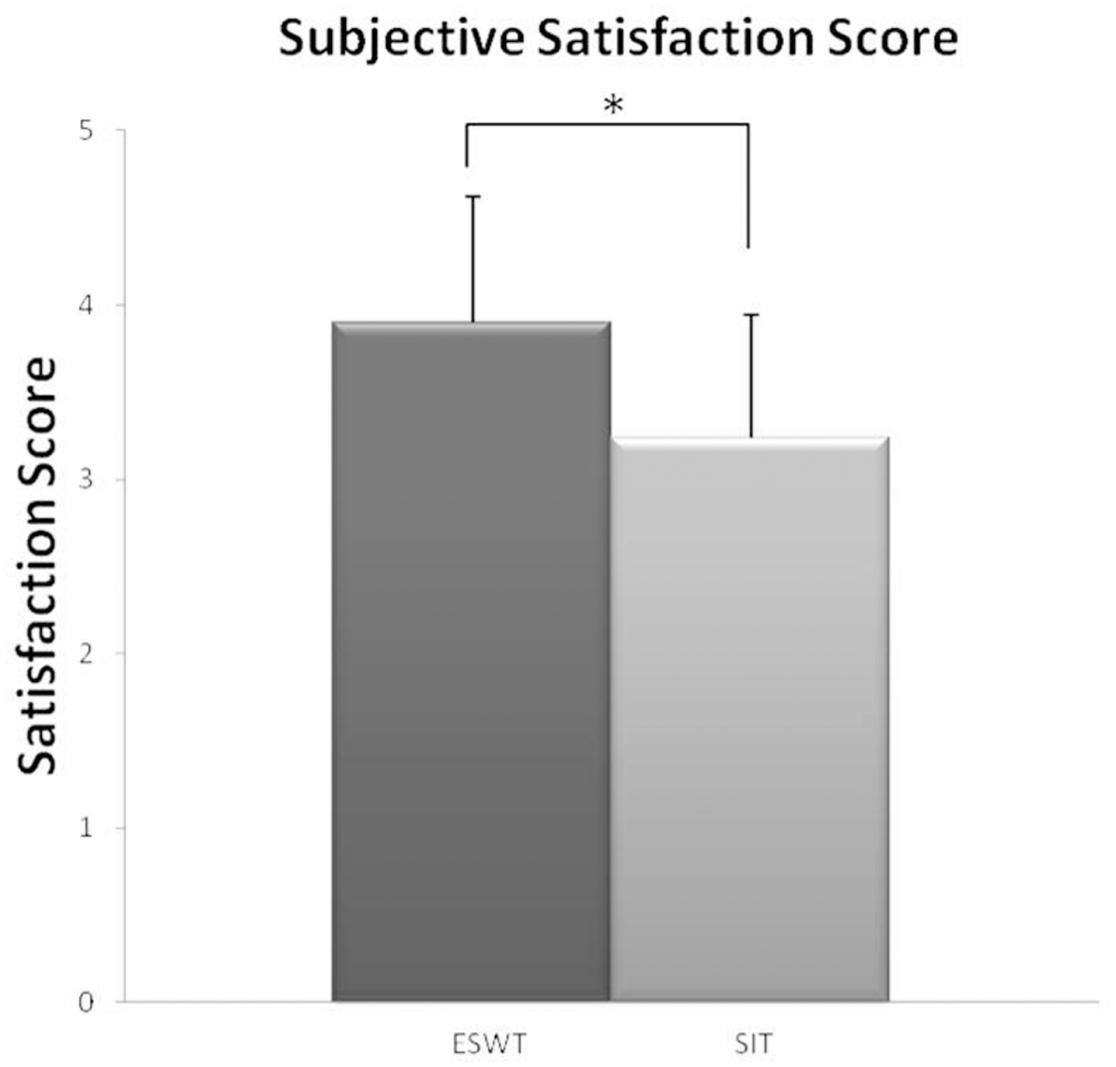
Patient satisfaction score. The subjective satisfaction score was higher in the ESWT group (*p < 0.01). Differences between the ESWT and SIT groups in the subjective satisfaction score at the 8^th^ week follow-up were analyzed by *t*-test / Wilcoxon rank-sum test. ESWT: mean±SD = 3.95±0.76 versus SIT group: mean±SD = 3.24±0.76, *t*-test p = 0.003/Wilcoxon rank-sum test p = 0.007.

To evaluate the subjective satisfaction after treatment, a 5-level scale was used for evaluation at the 8^th^ week after treatment. The excellent and good scores were categorized into one group, and the acceptable and poor scores were categorized into another group for comparison. The patients in the ESWT group had better subjective satisfaction scores, with 70% reporting good to excellent satisfaction. The scores were significantly higher in the ESWT group than in the SIT group (p = 0.003, Fig 2).

## Discussion

This study showed that non-invasive treatment options with ESWT and physical modalities with SWD and IFC were effective in reducing pain in coccydynia, and the effectiveness of ESWT was superior to the physical modalities, with regard to pain reduction, grade of improvement in disability, and subjective satisfaction.

ESWT was first used to disintegrate renal stones in 1980 [31], and it has also been used in the treatment of various musculoskeletal disorders. Studies have shown that ESWT could enhance tendon repair and decrease pain. The success rate for tendinopathy ranges from 60% to 80% [32]. However, the mechanism of ESWT in pain relief is not completely understood, and many theories have been proposed to explain the effects of ESWT. One proposed mechanism for the effectiveness of ESWT in tendinopathy is increased tissue regeneration through the induction of mechanotransduction on the cytoskeleton and the stimulation of protein synthesis [33]. ESWT facilitates tendon repair through up-regulating extracellular matrix biosynthesis and increasing the expression of TGFβ1 and IGF-I [34]. ESWT also decreases the levels of inflammatory mediators (interleukins and matrix metalloproteinases) [35] and promotes vascularization of the injured tendon junction [25] to promote tissue healing [36]. ESWT has an effect on pain transmission by acting on substance P [37]. ESWT can promote neovascularization by increasing the expression of factors including vascular endothelial growth factor (VEGF), endothelial nitric oxide synthase (eNOS), and proliferating cell nuclear antigen (PCNA) [25].

Patients with coccydynia show inflammatory changes in the area of the coccyx [8] and pain syndromes [9]. In a report evaluating the treatment and outcome analyses of patients with coccydynia, 66% of patients experienced significant improvement with conservative management using medication and/or local steroid injections [38]. Conservative treatments are generally recommended before surgical intervention with coccygectomy in the treatment of patients with coccydynia. Considering the effect of ESWT on reducing the inflammatory response, this non-invasive treatment modality might be beneficial for treating patients with coccydynia. There is only one recent report evaluating the effects of ESWT on pain relief in patients with coccydynia. Marwan et al. reported that three sessions of ESWT were effective in relieving pain, and the pain did not recur during a one-year follow-up period [27]. Our study randomized patients to ESWT or physical modality treatment to compare the treatment outcomes. In addition to pain evaluation, we included a disability rating scale for a more comprehensive evaluation of the treatment outcomes. The results suggested favorable outcomes in patients treated with ESWT regarding the degree of improvement in pain, disability and subjective satisfaction. We concluded that ESWT could be an alternative treatment option for patients with coccydynia and have a satisfactory treatment response. Patients receiving ESWT visited the hospital once each week for 4 weeks. Conversely, patients receiving physical modalities visited the hospital 3 times per week. The patients’ subjective satisfaction was better in the ESWT group, most likely because they had better treatment outcomes with fewer hospital visits and less time spent.

To determine the degree of functional improvement after different treatment modalities, the ODI score was evaluated in our study. The minimal clinically important difference (MCID) score is a key element in determining the response to specific treatment modalities [39]. However, there continues to be a lack of clarity for the suggested MCID score for the ODI [40]. In our study population, the initial scores suggested moderate disability in both the ESWT and SIT groups [29]. Regarding the percentage of change in the ODI score after treatment, both groups had scores with a more than 30% change at the 5^th^ week assessment, reaching a clinically meaningful improvement as reported by Ostelo et al [41]. At the 8^th^ week follow-up, the patients receiving ESWT showed further improvement in their disability scores, reaching a more than 50% change in the ODI score, a threshold improvement considered a successful outcome in low back pain patients as reported by Fritz et al [42]. This result suggested that ESWT provides a good treatment response for patients with coccydynia. The different recommended threshold improvements reported were mainly based on chronic low back pain populations; whether these thresholds are valid for the assessment of patients with coccydynia awaits further clinical studies.

There are some limitations to this study. The diverse etiologies of coccydynia should be studied with different treatments. The initial disability index differed between groups before therapy; however initial VAS scores were comparable between groups. The number of enrolled cases was not sufficiently large for etiological subgrouping, and further study with a larger number of cases is needed to compare ESWT with other treatment options, such as local steroid injections. Moreover, longitudinal follow-up over a longer period is indicated to evaluate the long-term effects of ESWT.

## Conclusions

In this study, ESWT appeared to be useful in relieving the pain of coccydynia and more effective in reducing pain syndromes than the use of physical modalities. Therefore, ESWT is recommended as an alternative method for treating patients with coccydynia.

## Supporting Information

S1 Text CONSORT 2010 checklist of information to include when reporting a randomised trial. (DOC)

S2 Text Original protocol. (PDF)

S3 Text Translated protocol. (PDF)
