# Supplementary material for: The Effects of Extracorporeal Shock Wave Therapy in Patients with Coccydynia: A Randomized Controlled Trial
Source: PLoS One. 2015 Nov 10;10(11):e0142475. doi: 10.1371/journal.pone.0142475 (PMC4640534; doi:10.1371/journal.pone.0142475)
Supplement: S2 Text — (PDF) [file pone.0142475.s002.pdf]

IRB 編號：KMUH-IRB-____________

（由承辦單位填寫）

※下列項目請詳細填寫

計畫名稱：

（中文）**「震波治療」對尾骨疼痛患者之療效：隨機控制試驗**

（英文）**Effect of Shockwave Therapy in patient with Coccydynia：A Randomized Control Trial**

研究成員	姓名	單位/職稱	聯絡電話/分機
計畫主持人	（中文）林士峰<br>（英文）Shih-Feng Lin	高雄市立大同醫院<br>復健中心物理治療師	07-2911101 # 8555
共同主持人	（中文）陳嘉炘<br>（英文）Chia-Hsin Chen	高雄市立大同醫院<br>復健科主任	07-2911101 # 8909

1.研究經費來源：

■無

□有，經費來源：

□國衛院 □中研院 □衛生署 □國科會 □高醫大 □高醫附院

□小港醫院 □大同醫院 □校際合作 □院際合作 □其它，____________

預計研究經費：____________

2.計畫執行場所（可複選）：

□高醫附院 □高醫大 □小港醫院 ■大同醫院 □其他：____________

3.研究目的及背景說明：

Coccydynia is a painful disorder of the tailbone (coccyx) localized just above the anus.[1] In the acute form of coccydynia, a trauma (usually a fall in the sitting position) is the cause of the complaints in the majority of the cases.[2, 3] Repetitive microtrauma resulting from an inadequate sitting posture or from activities such as cycling can also give rise to coccydynia.[4, 5] In females, parturition can be regarded as a trauma for the development of coccydynia.[6] The coccygeal joints are involved in 70% of traumatic childbirth cases.[2] Moreover, a relationship exists between weight and the occurrence of coccydynia; a body-mass index (BMI) of > 27.4 in females and > 29.4 in males increases the chance of developing coccydynia.[3]

Dynamic, radiological examination of function (coccyx stressed and unstressed) and discography indicates that the following five causes may play a role in these traumatic and idiopathic coccydynia: anterior luxation, hypermobility, coccygeal spicules, subluxation, and luxation.[2, 6] MRI studies show that mobility during tightening of the muscles of the pelvic floor and defecation is independent of age, gender, and the presence or absence of coccydynia. The coccyx usually consists of four bony segments that are attached cranially to the sacrum at the sacrococcygeal joint. Between the first two segments, a rudimentary intervertebral disc may be present and can form a potential localization point for post-traumatic hypermobility.[3] The other segments are synarthroses and have no mobility. Due to a more posteriorly situated sacrum and coccyx,[7] and a longer coccyx relative to men,[8] females have a greater chance of developing coccydynia. A clear relationship exists between coccydynia and the female gender; the female/male incidence ratio is 5:1.[9]

Treatment of coccydynia includes **conservative treatment** methods such as nonsteroidal anti-inflammatory drugs (NSAIDs), opioid drugs, gabapentin, pregabaline, myorelaxants, postural education, use of special cushion, physical therapy (massage, hot pack, electrical stimulation, and manual therapy),[8, 10-12] and **interventional treatment** methods such as local anesthetic, steroid injections, radiofrequency (RF) treatment.[8, 13, 14] Some patients may need **surgical treatment** such as coccygectomy.[15-17] In general, most treatments have been evaluated in retrospective studies, but there have been few controlled studies showing the efficacy of any known coccydynia treatments.

【Conservative Management】

In the acute phase of a post-traumatic coccydynia, a conservative policy has been proposed. This conservative approach includes NSAIDs and an adapted sitting posture.[8] In a controlled pilot study, conservative treatment, in the sense of mobilization of the coccyx, has been shown to have a long-term effect in 25% of patients.[10] A subsequent randomized controlled study, by the same group, compared intrarectal manipulation (applied in three 5-minute sessions over a period of 10 days) to shortwave magnetic field physiotherapy (delivered in three sessions over a period of 10 days). Intrarectal manipulation was more effective than the control treatment in improving visual analog scale (VAS) scores as well as functional and pain questionnaires. However, the efficacy was modest.[11] Infrared thermography before and after manual therapy and diathermy in patients with coccydynia objectively showed decrement of surface temperature correlating (r = 0.67, P < 0.01) with changes of subjective pain intensity after treatment.[12]

【Interventional Management】

In a prospective study, the combination of local injections of corticosteroids/local anesthetic with mobilization was shown to have a positive effect in 85% of cases, while local injections of corticosteroids/local anesthetic alone produced a 60% success rate.[1] The effect of intradiscal corticosteroid injections into the coccyx has yet to be demonstrated.[2] In addition to local injections of corticosteroids/local anesthetic, interventional pain management techniques include radiofrequency (RF) treatment of the sacral roots. The use of RF with a minimal invasive procedure for this group of patients as an alternative to surgical treatment and it might be an additional option among non-surgical treatment methods.[18] But further randomized prospective controlled studies in patients with coccydynia are needed to fully evaluate the effectiveness of RF.

【Surgical Management】

In the subacute and chronic phases, many forms of treatment for coccydynia are advised, up to and including surgical removal of the coccyx. Although retrospective studies are still being published concerning coccygectomy,[19] there are strong contraindications to this surgical intervention due to the long-term moderate results and the chance of major complications.[8]

In brief, interventional or surgical management always carries the risk of going through the disc and penetrating the rectum. Based on the results and complications reported, these treatments are not recommended for the principal treatment tools.[20]

【Shockwave therapy】

Some recent studies have shown that shockwave therapy is promising recovery in musculoskeletal disorders patients. The sources of shockwave generation include electrohydraulic, electromagnetic and piezoelectric principles. Electrohydraulic shockwaves are high-energy acoustic waves generated under water explosion with high voltage electrode. Shockwave in urology (lithotripsy) is primarily used to disintegrate urolithiasis, whereas shockwave in orthopedics (orthotripsy) is not used to disintegrate tissues, rather to induce tissue repair and regeneration.[21] The most important physical parameters of shockwave therapy for the treatment of orthopedic disorders include the pressure distribution, energy flux density and the total acoustic energy. In contrast to lithotripsy in which shockwaves disintegrate renal stones, orthopedic shockwaves are not being used to disintegrate tissue, but rather to microscopically cause interstitial and extracellular responses leading to tissue regeneration.[21] The application of shockwave therapy in musculoskeletal disorders has been around for more than a decade and is primarily used in the treatment of sports related over-use tendinopathies such as proximal plantar fasciitis of the heel, lateral epicondylitis of the elbow, calcific or non-calcific tendonitis of the shoulder and patellar tendinopathy etc.[22-37] The success rate ranged from 65% to 91%, and the complications were low and negligible. shockwave therapy is also utilized in the treatment of non-union of long bone fracture, avascular necrosis of femoral head, chronic diabetic and non-diabetic ulcers and ischemic heart disease.[38-45] The vast majority of the published papers showed positive and beneficial effects. FDA (USA) first approved shockwave therapy for the treatment of proximal plantar fasciitis in 2000 and lateral epicondylitis in 2002. Shockwave therapy is a novel non-invasive therapeutic modality without surgery or surgical risks, and the clinical application of shockwave therapy steadily increases over the years.

These findings imply the possibility of shockwave therapy in inducing tissue repair and regeneration in coccydynia patients. Therefore, a better understanding of the mechanism in recovery of coccydynia is a prerequisite for improving our rehabilitation services in the future. Although there are other interventional treatments relative to coccydynia, there is no shockwave therapy specifically focused on this disorder. However, to our knowledge, the effects of shockwave therapy on the underlying mechanisms responsible for recovery in coccydynia patients are not well understood. In the present study, we aimed to investigate the effectiveness of shockwave therapy in patients with coccydynia that could be more effect than conventional treatment protocols.

4.計畫內容（請以中文詳述①研究起訖期間、預定進度，②執行地點，③收案對象、樣本數，④執行步驟、方法，⑤納入條件（符合條件者，適合參加研究），⑥排除條件（符合條件者，不能參加研究），⑦研究設計、統計方法，⑧檢體採集方式、頻率、劑量等量化數據，⑨研究人力及相關設備需求）：

(1.) Sample：

We will recruit a total of 40 patients with a diagnosis of Coccydynia referred to Department of Physical Medicine & Rehabilitation of Kaohsiung Municipal Ta-Tung Hospital. Patients were randomly divided into 2 groups by assigning patients with an odd medical record number to the experimental group (20 patients) and patients with an even number to the control group (20 patients). Diagnosis was made from the chief complaint of pain and/or tenderness over the coccygeal area and the presence of pain when the coccyx was manipulated. Coccydynia was defined as pain in or around the coccyx, without any significant radiation, which is present mainly in the sitting position or when moving from the sitting to the standing position.[2]

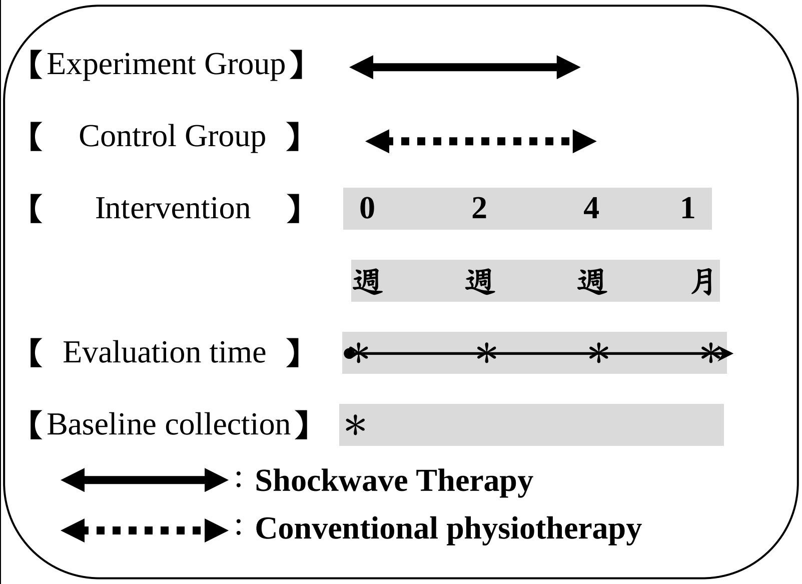

【Inclusion Criteria】

1.) Patients with chronic (>2 months) coccydynia (since, in our experience, many cases of acute coccydynia will remit spontaneously in under 2 months).

2.) Patients who were over 21 years and older and skeletally matured.

3.) Patients without ongoing antidepressant treatment

4.) Patients who have good knowledge to complete the questionnaire assessment.

5.) Patients who understood and complied with the nature of the study participation.

6.) Patients who agree to sign the informed consent form.

【Exclusion Criteria】

1.) Patients who received a cortisone injection within 6 weeks.

2.) Patients on immunosuppressant agents and/or corticosteroid within 6 months.

3.) Patients with diabetes mellitus, occlusive vascular disease, collagen disease, osteoarthritis or rheumatoid arthritis, coagulopathy, or infection.

4.) Patients with radiographic fractures around coccygeal area.

5.) Patients with neuropathic sign which could influence treatment effect.

6.) Patients with cardiac arrhythmia or cardiac pacemaker.

7.) Patients who were pregnant.

【Sample Size Estimation】

Because the sample size calculation for this study can't determine from the previous finding, An estimate of patients for each group required for a power of 80%, a one-sided type I error of 5%, and the dropout rate of 15% assumed from our experience with follow up evaluation of patients. Taken these considerations together, we plan to recruit 20 patients for each group to have an adequate sample size to test the proposed hypotheses**.**

(2.) Randomized Controlled Trial：

All the patients gave their informed consent, after which they were randomized to the experimental group or control group. Each patient was given a sealed envelope that bore his or her enrollment number and contained the treatment to which he or she was randomized. The letter was opened in the presence of the patient.

(3.) Treatment Protocol：

【Shockwave Therapy Group】

Patients in the study group received shockwave treatment as outpatients with no local or regional anesthesia. The source of shockwave was from the BTL-5000 series (BTL Industries Ltd; Hertfordshire, United Kingdom). Each patient was treated with the frequency of 10 Hz, 4 bar pressure, 2000 shocks, applied once a week, with a maximum of 4 treatments. The dosage was chosen based on our previous experience in shockwave application for other musculoskeletal disorders.[38-45] The point of maximal tenderness was elicited by palpation, and the location of the lesion was focused with the laser control guide of the device. The depth of treatment was estimated clinically and confirmed with an ultrasound guide. Surgical lubricant was applied to the skin in

contact with the shockwave tube. Treatment began with slow frequency at 1 impulse of shock per second and gradually increased to 2 shocks per second as the patient could tolerate the procedure. Any side effect within both groups will be recorded by the therapist.[46]

【Control Group】

Patients in the control group were treated with conventional physiotherapy including short-wave diathermy and interference current therapy (IFC). The patient is asked to lay prone with the lower back and buttocks exposed. A pelvic pillow support was given. Firstly, a qualified therapist applied continuous short-wave diathermy using the machine (SW50, Cosmogamma, Italy). The hinge-type drum of the inductive electrode was used with 2 lateral sections containing the actual electrodes and the central unit consisting of the lead connections only. The drums were spread widely and placed so that the entire coccygeal area of the patient was in the contact field. A towel was placed under the drum to separate the lead wires from the patient's skin. The short-wave diathermy treatment was carried out for 20 minutes.[12] Secondly, the therapist administered the IFC which was a 4 KHz sinusoidal biphasic electric current with amplitude that was modulated between 60 and 100 Hz, with a ramp and fall of one second each and constant phases of two seconds in between to the subject who keep the same position as short-wave diathermy.[29, 30, 36, 47] For each patient two paired reusable hypoallergic vacuum circular electrodes were used (Minato Inc, SK-9S, Japan). Four electrodes were placed widely in order to cover the entire coccygeal area. This electrotherapeutical concept is commonly recommended and used.[48] The position of all electrodes was kept fixed during all treatment sessions for 20 minutes. Hence, the control group accepts totally 40 minutes conventions treatment in each session, the 3 sessions per week, for 4 weeks.

(4.) Outcome Measures：

All the subjects' demographic characteristics and related measurements were recorded before the experiment include age, gender, body mass index (BMI), duration of the complaints, and traumatic or nontraumatic etiology.

Primary outcome variables for this study will be the visual analogue scale (VAS)[11, 12, 18] and the Oswestry Disability Index (ODI).[49] The first subjective assessment of pain intensity was analyzed applying visual analogue scale (VAS). The VAS is used to measure pain on a 100 mm horizontal axis between the extreme left endpoint of no coccyx pain and the extreme right endpoint of the worst pain. The distance is then measured and pain is recorded on a 100-point scale.[11, 12, 18] The second subjective assessment of pain intensity was Oswestry Disability Index version 2.1 (also

known as the Oswestry Low Back Pain Disability Questionnaire) which is an extremely important tool that researchers and disability evaluators use to measure a patient's permanent functional disability. The test is considered the “gold standard” of low back functional outcome tools. There are totally 10 sections within ODI. For each section the total possible score is 5: if the first statement is marked the section score = 0; if the last statement is marked, it = 5. The percentage of the total scores divided by total possible score means level of disability (0% to 20%：Minimal disability；21%-40%：Moderate disability；41%-60%：Severe disability；61%-80%：Crippled；81%-100%：Bed-bound).[49]

The clinical measures will be administered to patients by the same blinded rater at pre intervention, 2 weeks intervention, post intervention, 1-month follow-up. Prior to administration of clinical measures, the blinded rater will be trained to properly administer these measures. The assessor was also blinded to know each other’s group during the study period.

(5.) Data analysis

In this study, repeated measures analysis of variance (ANOVA), treating time as a within subject factor and group as a between-subject factor, followed by post hoc multiple comparisons were used to evaluate treatment efficacy with group as the between-subjects factor and scores of the visual analogue scale (VAS) and the Oswestry Disability Index (ODI) as the dependent variables. To take into consideration the effect of multiple testing, the Sharpened Bonferroni method was used to adjust for individual alpha level, while the overall level of significance was set at 0.05.

(6.) Achieved percentage of scheduled progress

Items \ Time	2013				2014
	1m~3m	4m ~6m	7m ~9m	10m ~12m	1m~3m
Prepared					
Recruit subject (No.1~20)					
Effective assessment					
**Poster** presentation					
Recruit subject (No.20~40)					
Effective assessment					
Data analysis (No.1~40)					
**Oral** presentation					
Paper publish					
**percentage of progress** (%)	25	50	75	100	

5.預期試驗效果及其效益：

(1.) Anticipated Results

When the study is completed, there will be some breakthroughs in the field of shockwave therapy in coccydynia. This will be the first study comprehensively investigating the effects of shockwave therapy in patients with coccydynia. According to regaining functional activity on decreasing pain based on clinical scales such as VAS, ODI scores, the effectiveness of optimal therapeutic parameter settings will be evaluated. The results of this study will help to clarify the effects and suitability of shockwave intervention, and keep our domestic research team the leader position in this field. After completing this project, we will continue to promote the function of shockwave treatment system. Based on above-mentioned results and the evidence-based combination of parameters, we will be in a pole position to further investigate the feasibility of the program for shockwave usage.

(2.) Training effect of staff

The researcher can familiar with treatment protocol and operation of shockwave therapy, understanding the process of recovery in patient with coccydynia. That clinician can improve their technique using on other patient with coccydynia. Moreover, the rater will well-known about those assessment tools.

6.可能發生之副作用與危險及其處理方式：

Shockwave therapy is a novel non-invasive therapeutic modality without surgery or surgical risks, and the clinical application of shockwave therapy steadily increases over the years. Although FDA (USA) have been approved shockwave therapy for the treatment of proximal plantar fasciitis first in 2000 and lateral epicondylitis in 2002, all participate still be inspected for swelling, ecchymosis, and hematoma by clinical doctor at every session after shockwave treatment. Postoperative management included ice pack to the treatment site and a prescription of nonnarcotic analgesic, such as acetaminophen, when subject have any side effect during the intervention management. Patients were allowed to resume light activity; however, heavy activities including sports were not permitted and following by clinical doctor for 4 to 6 weeks.

7.受試者招募方式：

■口頭介紹、說明

□海報（請檢附審查）

□網路（請檢附內容審查）

□其他：________________

□不適用

8.詳述本研究之醫學倫理規範：

We propose to recruit about 40 eligible patients with coccydynia during the study period. Clinical information about the patients will be obtained from medical records. Therefore, an invitation letter will be sent out to obtain written informed consent from each participant. The letter will explain plainly and in simple terms to address the purposes of the study, the procedures, and the risks and benefits, as well as the processes of informed consent in this project. In addition, we will obtain the approval of the IRB (Institution Review Board) of the Kaohsiung Medical University Chung-Ho Memorial Hospital. The results of this project will remain confidential. Furthermore, the participants' data will be kept in locked file cabinets located in research office, accessible only to the study personnel.

9.受試者權益與檢體使用者之義務：

① 檢體處理方式及儲存地點

□________________________________

□不適用

② 檢體是否輸出至境外使用？

□是，單位：____________________

■否

□不適用

10.是否提供受試者同意書給受試者：

※ 請自行勾選下列項目，但相關之隱私保護與是否同意免除受試者同意書，其最後裁定權為本院人體試驗審查委員會

■是

□申請免除受試者同意書，免除理由：____

**11.研發成果之歸屬及運用**<br>(1) 本計畫研究成果將發表於學術期刊。<br>(2) 如本計畫研究成果獲得學術文獻發表、智慧財產及實質效益時，您同意無償贈與高雄醫學大學/高雄醫學大學附設中和紀念醫院/高雄市立小港醫院/高雄市立大同醫院作為從事疾病診斷、預防、治療及研究等醫學用途。
**12.主持人聲明：**<br>(1)本人負責執行本研究計畫。願依赫爾辛基宣言的精神及國內相關法令的規定，確保試驗對象之生命、個人隱私及尊嚴。<br>(2)若以後有任何計畫內容的修改，除了要立即降低危險性的情況外，在未獲得本院人體試驗審查委員會同意前，絕不會進行修改後的內容。<br>計畫主持人簽章：林士峰　　西元 2012 年 09 月 02 日<br>共同主持人簽章：陳嘉炘、謝清麟　　西元 2012 年 09 月 02 日<br>協同主持人簽章：　　西元　年　月　日

※本表若不敷使用，請自行延伸使用

References：

1. Wray CC, Easom S, Hoskinson J. Coccydynia. Aetiology and treatment. *The Journal of bone and joint surgery. British volume*. 1991;73:335-338
2. Maigne JY, Guedj S, Straus C. Idiopathic coccygodynia. Lateral roentgenograms in the sitting position and coccygeal discography. *Spine*. 1994;19:930-934
3. Maigne JY, Doursounian L, Chatellier G. Causes and mechanisms of common coccydynia: Role of body mass index and coccygeal trauma. *Spine*. 2000;25:3072-3079
4. Frazier LM. Coccydynia: A tail of woe. *North Carolina medical journal*. 1985;46:209-212
5. Sluijter ME. Percutaneous intradiscal radio-frequency thermocoagulation. *Spine*. 1996;21:528-529
6. Maigne JY, Tamalet B. Standardized radiologic protocol for the study of common coccygodynia and characteristics of the lesions observed in the sitting position. Clinical elements differentiating luxation, hypermobility, and normal mobility. *Spine*. 1996;21:2588-2593
7. Ng CL. Levator ani syndrome - a case study and literature review. *Australian family physician*. 2007;36:449-452
8. De Andres J, Chaves S. Coccygodynia: A proposal for an algorithm for treatment. *The journal of pain : official journal of the American Pain Society*. 2003;4:257-266
9. Peyton FW. Coccygodynia in women. *Indiana medicine : the journal of the Indiana State Medical Association*. 1988;81:697-698
10. Maigne JY, Chatellier G. Comparison of three manual coccydynia treatments: A pilot study. *Spine*. 2001;26:E479-483; discussion E484
11. Maigne JY, Chatellier G, Faou ML, Archambeau M. The treatment of chronic coccydynia with intrarectal manipulation: A randomized controlled study. *Spine*. 2006;31:E621-627
12. Wu CL, Yu KL, Chuang HY, Huang MH, Chen TW, Chen CH. The application of infrared thermography in the assessment of patients with coccygodynia before and after manual therapy combined with diathermy. *Journal of manipulative and physiological therapeutics*. 2009;32:287-293
13. Fogel GR, Cunningham PY, 3rd, Esses SI. Coccygodynia: Evaluation and management. *The Journal of the American Academy of Orthopaedic Surgeons*. 2004;12:49-54
14. Mitra R, Cheung L, Perry P. Efficacy of fluoroscopically guided steroid injections in the management of coccydynia. *Pain physician*. 2007;10:775-778
15. Capar B, Akpinar N, Kutluay E, Mujde S, Turan A. [coccygectomy in patients with coccydynia]. *Acta orthopaedica et traumatologica turcica*. 2007;41:277-280
16. Wood KB, Mehbod AA. Operative treatment for coccygodynia. *Journal of spinal disorders & techniques*. 2004;17:511-515

17. Balain B, Eisenstein SM, Alo GO, Darby AJ, Cassar-Pullicino VN, Roberts SE, Jaffray DC. Coccygectomy for coccydynia: Case series and review of literature. *Spine*. 2006;31:E414-420
18. Atim A, Ergin A, Bilgic S, Deniz S, Kurt E. Pulsed radiofrequency in the treatment of coccygodynia. *Agri*. 2011;23:1-6
19. Traub S, Glaser J, Manino B. Coccygectomy for the treatment of therapy-resistant coccygodynia. *Journal of surgical orthopaedic advances*. 2009;18:147-149
20. Albrektsson B. Sacral rhizotomy in cases of ano-coccygeal pain. A follow-up of 24 cases. *Acta orthopaedica Scandinavica*. 1981;52:187-190
21. Ogden JA, Toth-Kischkat A, Schultheiss R. Principles of shock wave therapy. *Clinical orthopaedics and related research*. 2001:8-17
22. Buch M, Knorr U, Fleming L, Theodore G, Amendola A, Bachmann C, Zingas C, Siebert WE. [extracorporeal shockwave therapy in symptomatic heel spurs. An overview]. *Der Orthopade*. 2002;31:637-644
23. Perez M, Weiner R, Gilley JC. Extracorporeal shock wave therapy for plantar fasciitis. *Clinics in podiatric medicine and surgery*. 2003;20:323-334
24. Roehrig GJ, Baumhauer J, DiGiovanni BF, Flemister AS. The role of extracorporeal shock wave on plantar fasciitis. *Foot and ankle clinics*. 2005;10:699-712, ix
25. Strash WW, Perez RR. Extracorporeal shockwave therapy for chronic proximal plantar fasciitis. *Clinics in podiatric medicine and surgery*. 2002;19:467-476
26. Thomson CE, Crawford F, Murray GD. The effectiveness of extra corporeal shock wave therapy for plantar heel pain: A systematic review and meta-analysis. *BMC musculoskeletal disorders*. 2005;6:19
27. Wilner JM, Strash WW. Extracorporeal shockwave therapy for plantar fasciitis and other musculoskeletal conditions utilizing the ossatron--an update. *Clinics in podiatric medicine and surgery*. 2004;21:441-447, viii
28. Buchbinder R, Green SE, Youd JM, Assendelft WJ, Barnsley L, Smidt N. Systematic review of the efficacy and safety of shock wave therapy for lateral elbow pain. *The Journal of rheumatology*. 2006;33:1351-1363
29. Rompe JD, Theis C, Maffulli N. [shock wave treatment for tennis elbow]. *Der Orthopade*. 2005;34:567-570
30. Rompe JD, Maffulli N. Repetitive shock wave therapy for lateral elbow tendinopathy (tennis elbow): A systematic and qualitative analysis. *British medical bulletin*. 2007;83:355-378
31. Stasinopoulos D, Johnson MI. Effectiveness of extracorporeal shock wave therapy for tennis elbow (lateral epicondylitis). *British journal of sports medicine*. 2005;39:132-136

32. Mouzopoulos G, Stamatakos M, Mouzopoulos D, Tzurbakis M. Extracorporeal shock wave treatment for shoulder calcific tendonitis: A systematic review. *Skeletal radiology*. 2007;36:803-811
33. Spindler A, Berman A, Lucero E, Braier M. Extracorporeal shock wave treatment for chronic calcific tendinitis of the shoulder. *The Journal of rheumatology*. 1998;25:1161-1163
34. van Leeuwen MT, Zwerver J, van den Akker-Scheek I. Extracorporeal shockwave therapy for patellar tendinopathy: A review of the literature. *British journal of sports medicine*. 2009;43:163-168
35. Rasmussen S, Christensen M, Mathiesen I, Simonson O. Shockwave therapy for chronic achilles tendinopathy: A double-blind, randomized clinical trial of efficacy. *Acta orthopaedica*. 2008;79:249-256
36. Furia JP. High-energy extracorporeal shock wave therapy as a treatment for chronic noninsertional achilles tendinopathy. *The American journal of sports medicine*. 2008;36:502-508
37. Vulpiani MC, Vetrano M, Savoia V, Di Pangrazio E, Trischitta D, Ferretti A. Jumper's knee treatment with extracorporeal shock wave therapy: A long-term follow-up observational study. *The Journal of sports medicine and physical fitness*. 2007;47:323-328
38. Cacchio A, Giordano L, Colafarina O, Rompe JD, Tavernese E, Ioppolo F, Flamini S, Spacca G, Santilli V. Extracorporeal shock-wave therapy compared with surgery for hypertrophic long-bone nonunions. *The Journal of bone and joint surgery. American volume*. 2009;91:2589-2597
39. Elster EA, Stojadinovic A, Forsberg J, Shawen S, Andersen RC, Schaden W. Extracorporeal shock wave therapy for nonunion of the tibia. *Journal of orthopaedic trauma*. 2010;24:133-141
40. Xu ZH, Jiang Q, Chen DY, Xiong J, Shi DQ, Yuan T, Zhu XL. Extracorporeal shock wave treatment in nonunions of long bone fractures. *International orthopaedics*. 2009;33:789-793
41. Hyer CF, Vancourt R, Block A. Evaluation of ultrasound-guided extracorporeal shock wave therapy (eswt) in the treatment of chronic plantar fasciitis. *The Journal of foot and ankle surgery : official publication of the American College of Foot and Ankle Surgeons*. 2005;44:137-143
42. Ibrahim MI, Donatelli RA, Schmitz C, Hellman MA, Buxbaum F. Chronic plantar fasciitis treated with two sessions of radial extracorporeal shock wave therapy. *Foot & ankle international / American Orthopaedic Foot and Ankle Society [and] Swiss Foot and Ankle Society*. 2010;31:391-397
43. Kudo P, Dainty K, Clarfield M, Coughlin L, Lavoie P, Lebrun C. Randomized, placebo-controlled, double-blind clinical trial evaluating the treatment of plantar fasciitis with an extracoporeal shockwave therapy (eswt) device: A north american confirmatory

study. *Journal of orthopaedic research : official publication of the Orthopaedic Research Society*. 2006;24:115-123

44. Kikuchi Y, Ito K, Ito Y, Shiroto T, Tsuburaya R, Aizawa K, Hao K, Fukumoto Y, Takahashi J, Takeda M, Nakayama M, Yasuda S, Kuriyama S, Tsuji I, Shimokawa H. Double-blind and placebo-controlled study of the effectiveness and safety of extracorporeal cardiac shock wave therapy for severe angina pectoris. *Circulation journal : official journal of the Japanese Circulation Society*. 2010;74:589-591
45. Sheu JJ, Sun CK, Chang LT, Fang HY, Chung SY, Chua S, Fu M, Lee FY, Kao YH, Ko SF, Wang CJ, Yen CH, Leu S, HK Y. Shockwave-pretreatedbonemarrowcells further improveleftventricularfunctionaftermyocardialinfarction in rabbits. *Annals of Vascular Surgery*. 2010;24:809-821
46. Wang CJ, Ko JY, Chan YS, Weng LH, Hsu SL. Extracorporeal shockwave for chronic patellar tendinopathy. *The American journal of sports medicine*. 2007;35:972-978
47. Sato A, Schmidt RF. Somatosympathetic reflexes: Afferent fibers, central pathways, discharge characteristics. *Physiological reviews*. 1973;53:916-947
48. Van Der Heijden GJ, Leffers P, Wolters PJ, Verheijden JJ, van Mameren H, Houben JP, Bouter LM, Knipschild PG. No effect of bipolar interferential electrotherapy and pulsed ultrasound for soft tissue shoulder disorders: A randomised controlled trial. *Annals of the rheumatic diseases*. 1999;58:530-540
49. Hodges SD, Eck JC, Humphreys SC. A treatment and outcomes analysis of patients with coccydynia. *The spine journal : official journal of the North American Spine Society*. 2004;4:138-140
